# Combined pulse radiofrequency and selective nerve root block for lumbar disc herniation-related neuropathic pain: a retrospective cohort study

**DOI:** 10.3389/fmed.2026.1777586

**Published:** 2026-03-18

**Authors:** Jie Chen, Hui Lu, Xinchao Jiang, Yi Song, Bin Qian, Mei Fang, Jianxue Qian, Cailin Wang

**Affiliations:** 1 Department of Orthopedic Surgery, First Affiliated Hospital of Soochow University, Suzhou, China; 2 Department of Pain, Suzhou Hospital of Integrated Traditional Chinese and Western Medicine, Suzhou, China; 3 Medical Research Center, The People’s Hospital of Suzhou New District, Suzhou, China

**Keywords:** dexamethasone palmitate, lumbardisc herniation, minimally invasive therapy, nerve root infiltration, neuropathic pain, pulse radiofrequency

## Abstract

**Objective:**

Compare the clinical efficacy and safety of pulse radiofrequency combined with selective nerve root block using dexamethasone palmitate and oral medication in the treatment of neuropathic pain caused by lumbar disc herniation.

**Methods:**

Retrospective analysis of 557 patients with lumbar disc herniation admitted to Suzhou Hospital of Integrated Traditional Chinese and Western Medicine from January 2019 to December 2024, including 208 males and 349 females, with an average age of 55.37 ± 13.70 years old. 342 cases were treated with oral diclofenac sodium (Control group), and 215 cases were treated with selective nerve root block using dexamethasone palmitate (Intervention group). Compare the clinical efficacy indicators such as VAS score, ODI score, JOA score, and modified MacNab score between two groups of patients before and after treatment.

**Results:**

There was no statistically significant difference in general information such as gender, age, disease course, and lesion segment between the two groups of patients (*P* > 0.05). Similarly, there was no significant statistical difference in preoperative VAS score, ODI score, and JOA score between the two groups of patients (*P* > 0.05). The VAS scores of the Control group at 1 week, 1 month, 6 months, and 12 months after surgery were 3.07 ± 1.326, 3.67 ± 0.933, 4.18 ± 1.444, 4.23 ± 1.407, the postoperative ODI score was 22.12 ± 2.871, and the postoperative JOA score was 16.25 ± 2.856; The VAS scores of the Intervention group at 1 week, 1 month, 6 months, and 12 months postoperatively were 2.15 ± 0.951, 1.80 ± 0.730, 2.07 ± 0.979, 2.25 ± 0.947, and the postoperative ODI score was 14.24 ± 1.990. The postoperative JOA score was 22.58 ± 2.132, and the differences were statistically significant (*P* < 0.05). The Excellent and Good rate of postoperative modified MacNab Score in the Intervention group was significantly higher than that in the Control group (*P* < 0.05). No serious complications or drug side effects were observed in both groups of patients.

**Conclusion:**

Compared with oral medication treatment, pulse radiofrequency combined with selective nerve root block using dexamethasone palmitate can more effectively alleviate neuropathic pain caused by lumbar disc herniation, improve quality of life, and promote lumbar functional recovery.

## Introduction

Lumbar disc herniation (LDH) stands as one of the most prevalent degenerative spine conditions, topping the list of spinal canal diseases in terms of incidence (1–4). This condition arises when the annulus fibrosus relaxes or ruptures, causing the nucleus pulposus to protrude or extrude and compress the spinal cord or nerve roots posteriorly (5). LDH patients subsequently experience neuropathic pain symptoms, such as low back pain, leg pain, and numbness (6–8). In more severe instances, sexual dysfunction or bladder and bowel dysfunction may manifest (2, 9–11), severely hampering patients’ ability to work and greatly affecting their quality of life.

Currently, LDH treatment encompasses a spectrum of options, ranging from conservative therapy to minimally invasive interventional therapy and surgical treatment. While conservative treatment offers a non-invasive approach, it is often characterized by a prolonged duration, recurrent symptoms, and limited pain relief in the short term (12, 13). Surgical treatment, though effective, is associated with high costs, significant invasiveness, and a risk of postoperative complications (14–19). Given these considerations, minimally invasive interventional therapy has emerged as a pivotal treatment modality for LDH, offering a balance between efficacy and patient safety (20–24). In the realm of minimally invasive interventional techniques, Pulse Radiofrequency (PRF) has gained traction due to its minimal trauma and swift recovery profile (25, 26). PRF effectively alleviates nerve root compression symptoms while safeguarding nerve and spinal function (27, 28). Recognizing the complexity of LDH’s clinical manifestations, our hospital has innovatively combined PRF guided by digital subtraction angiography (DSA) with selective nerve root block using dexamethasone palmitate (29–32). This combined approach were associated with improved outcomes, addressing the limitations of single minimally invasive therapies and enhancing therapeutic efficacy. The detailed results of this innovative treatment regimen are reported as follows.

## Materials and methods

### General information

**FIGURE 1:**
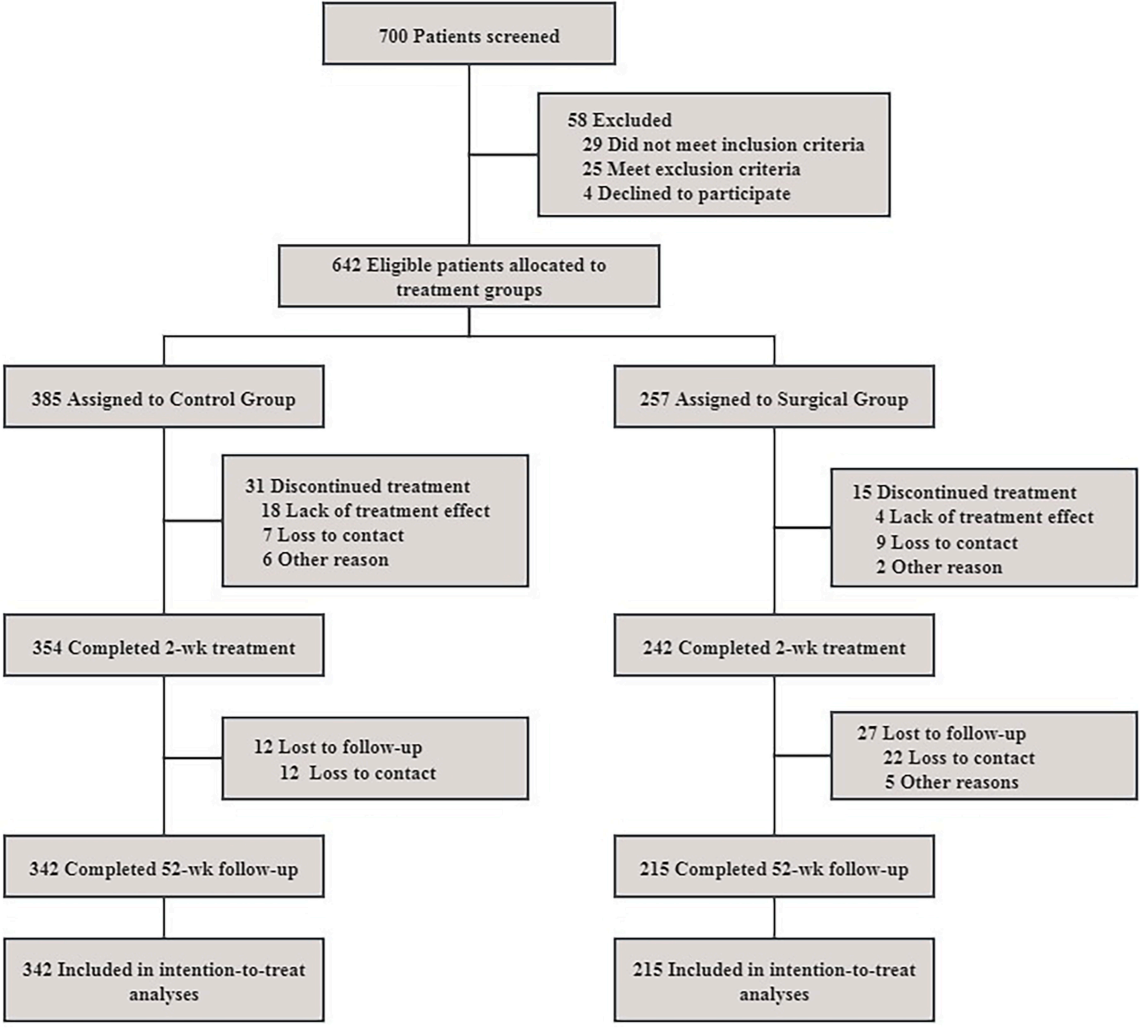
Flowchart comparing the therapeutic effects of lumbar disc herniation.

This study collected 700 patients with neuropathic pain caused by LDH who received treatment in Suzhou Hospital of Integrated Traditional Chinese and Western Medicine from January 2019 to December 2024. Patients were retrospectively categorized into a Control group and an Intervention group based on the treatment received during routine clinical care. Ultimately, 342 patients were included in the Control group and 215 in the Intervention group for statistical analysis (Figure 1). This study was approved by the Medical Ethics Committee of Suzhou Hospital of Integrated Traditional Chinese and Western Medicine.

### Inclusion and exclusion criteria

Inclusion Criteria: 1. Diagnosis based on the diagnostic criteria for Lumbar Disc Herniation, with typical symptoms of low back and leg pain, and sensory or motor dysfunction in one or both lower limbs; 2. Positive straight leg raising test and Bragard test; 3. Symptoms and signs consistent with the imaging examination findings in terms of the affected spinal segment; 4. No significant improvement after conservative treatment for more than 3 months; 5. Patients and/or their families have given consent and signed the informed consent form.

Exclusion criteria: 1. Lower limb symptoms and signs inconsistent with the herniated segment; 2. History of spinal surgery; 3. Severe compression of the cauda equina due to moderate/severe spinal stenosis, lumbar spondylolisthesis, ossification of the posterior longitudinal ligament, lumbar disc extrusion, etc.; 4. Inability to undergo surgery due to severe medical conditions such as hypertension, hyperglycemia, or coagulation disorders; 5. Lower limb symptoms caused by lumbar infections, tuberculosis, tumors, etc.; 6. Inability to cooperate with treatment due to psychological or psychiatric disorders.

### Treatment methods

Preoperative preparation: Actively manage underlying conditions such as blood pressure and blood sugar. Fasting and water restriction for 4 h before surgery. Skin preparation and surgical marking of the operative area.

**FIGURE 2:**
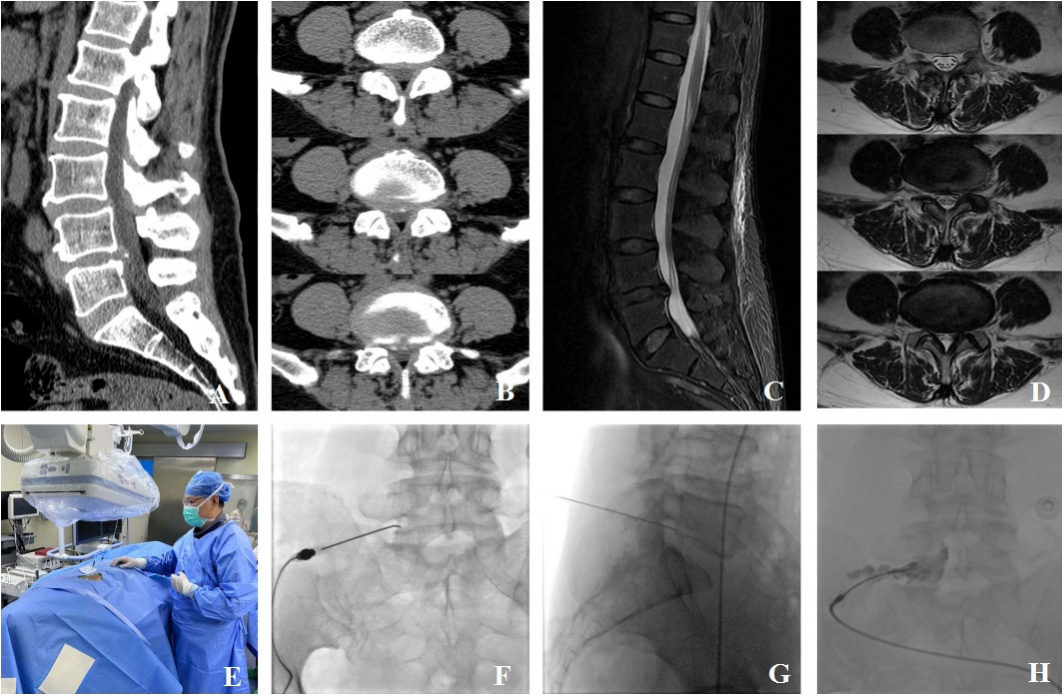
A typical case of DSA-guided radiofrequency pulsing combined with selective nerve root block. The patient is a 59-year-old female who was admitted due to “lumbosacral pain with numbness in the right lower limb for 4 months.” Lumbar spine CT scans in sagittal **(A)** and axial **(B)** planes revealed an L4/5 disc herniation (central-paracentral type) with calcification, right lateral recess stenosis, and posterior dural sac compression. Lumbar spine MRI scans in sagittal **(C)** and axial **(D)** planes also demonstrated an L4/5 disc herniation (central-paracentral type) with local subcutaneous soft tissue edema. **(E)** Intraoperative image of the DSA-guided radiofrequency pulsing procedure. **(F,G)** The radiofrequency needle puncturing under DSA guidance to reach the right L5/S1 intervertebral foramen. **(H)** A post-radiofrequency pulsing treatment image with iohexol contrast, showing the contrast agent traveling along the spinal nerve and within the vertebral canal.

Surgical method: (Taking the left L5 spinal nerve as an example) The patient is positioned prone with a soft pillow under the abdomen. After routine skin disinfection and draping, the puncture point is determined under DSA fluoroscopy as the left L5/S1 intervertebral foramen. A radiofrequency (RF) needle is inserted under DSA fluoroscopy, and DSA is used again to confirm that the needle tip is located within the intervertebral foramen. The needle core is then removed, and an RF electrode is inserted. RF testing is initiated, with a sensory test at 0.2∼0.4 V to induce pain at the lesion site and a motor test at 0.4∼0.8 V to induce muscle contraction at the lesion site. The pulsed RF mode is activated, with a cycle of 42°C for 120 s, repeated for a total of three cycles. Upon completion of RF treatment, the RF electrode is removed, and 0.5 mL of iohexol contrast agent is injected. DSA fluoroscopy reveals that the contrast agent travels along the spinal nerve and within the spinal canal. A total of 1.0 mL of analgesic composite solution (consisting of 2 mL of 2% lidocaine, 2 mL of 0.9% saline, and 1 mL of dexamethasone palmitate injection) is injected. After the procedure, the puncture needle is removed, mild pressure is applied to the wound, and a sterile dressing is applied. The patient is observed for 10 min, and upon confirming stable vital signs, they are safely returned to the ward (Figure 2).

Postoperative management: After surgery, the patient is advised to rest in bed primarily and undergo electrocardiographic monitoring for 6 h. When ambulating, the patient should wear lumbar supports such as a waist belt. Additionally, the patient is guided through functional rehabilitation training for the lower limbs and is advised to avoid engaging in heavy physical labor within 3 months after surgery.

Control group: Patients in the Control group receive slow-release diclofenac sodium tablets, 0.1 g, once daily (QD), orally, for a treatment duration is no less than 2 weeks.

### Evaluation indicators

Primary outcome indicators: 1. VAS Score: To assess the degree of pain relief in patients. 2. ODI Score: To evaluate the impact of lumbar disc herniation on normal life before and after treatment. 3. JOA Score: To assess the improvement of lumbar function.

Secondary outcome indicators: 1. Modified MacNab Score: To evaluate the clinical efficacy. 2. Surgical complications and drug side effects, including postoperative upper gastrointestinal discomfort, pain at the puncture site, worsening pain, sensory loss, new onset of numbness in the lower limbs, bleeding, infection, hypotension of cranial cavity, etc.

### Statistical analysis

The statistical software SPSS 20.0 was used for analysis. The Shapiro-Wilk test was employed to check the normality of the data. For normally distributed measurement data, they were expressed as mean ± standard deviation ($\bar{x}$ ± s), and comparisons between groups were conducted using the independent two-sample *t*-test. For count data, they were presented in terms of cases and/or percentages, and comparisons between groups were made using the chi-square (*χ^2^*) test. A *P*-value less than 0.05 was considered statistically significant, indicating a difference that was statistically meaningful.

## Results

### Comparison of general information

A comparison of general information such as gender, age, duration of illness, and lesion segments between the two groups revealed no statistically significant differences (*P* > 0.05), indicating that the two groups were comparable (Table 1).

**TABLE 1:** Comparison of general information between two groups of patients.

Parameters	Total (*n* = 557)	Control group (*n* = 342)	Intervention group (*n* = 215)	*P*-value	95% CI
Sex, n(%)				0.258	0.860–1.751
Male	208(37.34)	134(39.18)	74(34.42)		
Female	349(62.66)	208(60.82)	141(65.58)		
Age, year	55.37 ± 13.70	54.17 ± 11.75	56.69 ± 15.57	0.326	–7.594 to 2.546
Course of disease, week	189.30 ± 30.48	202.78 ± 40.60	174.58 ± 46.14	0.646	–93.108 to 149.511
Pathological segment, n(%)				0.894	0.882–0.931
L1/2	8(1.44)	5(1.46)	3(1.40)		
L2/3	34(6.10)	23(6.73)	11(5.12)		
L3/4	102(18.31)	60(17.54)	42(19.53)		
L4/5	209(37.52)	131(38.30)	78(36.28)		
L5/S1	204(36.62)	123(35.96)	81(37.67)		

### Comparison of VAS, ODI, and JOA scores

**FIGURE 3:**
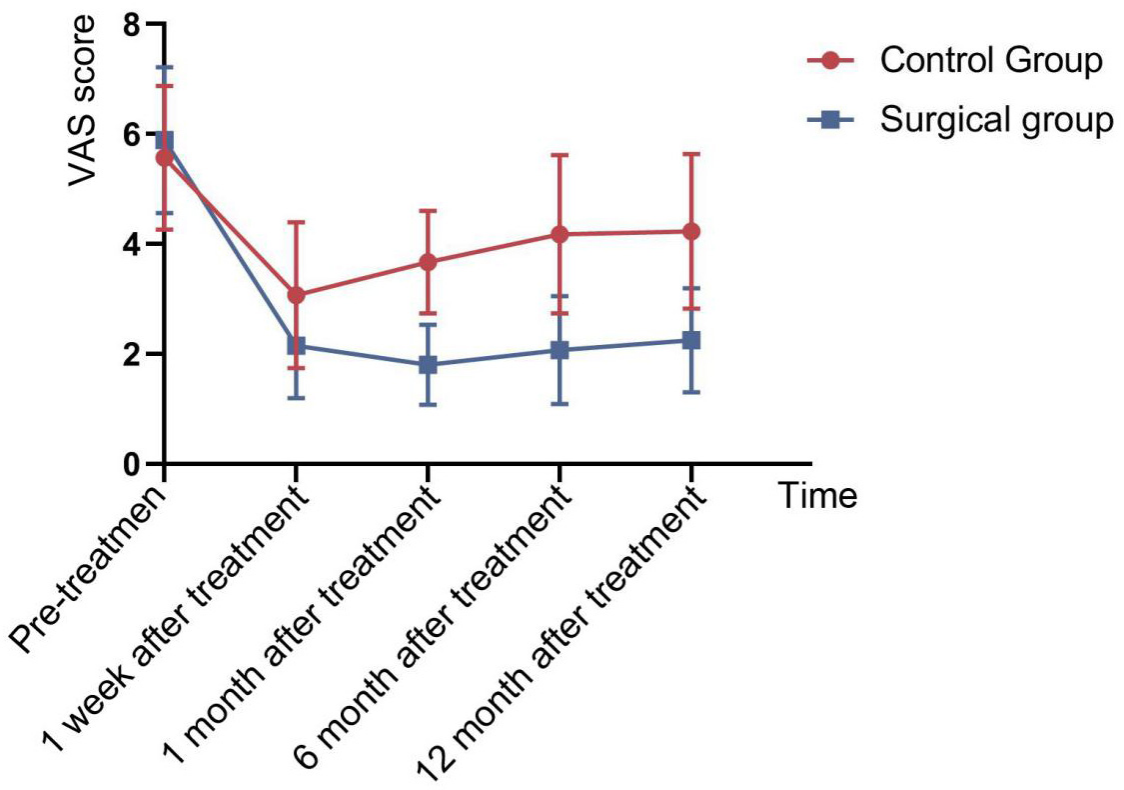
Comparison of VAS scores at different time points postoperatively between the two groups.

**FIGURE 4:**
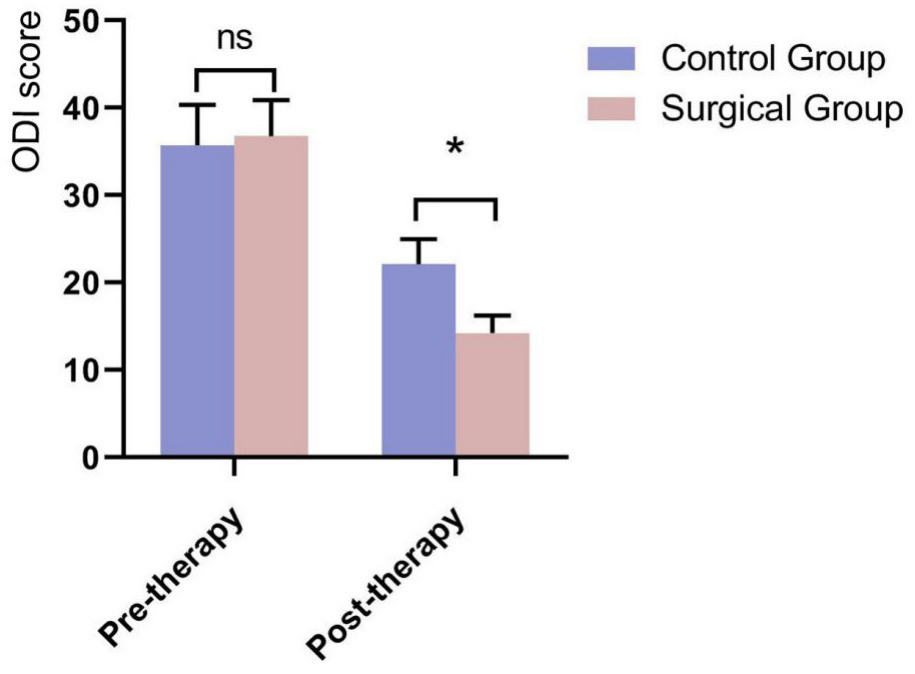
Comparison of ODI scores 1 week postoperatively between the two groups. *Indicates that the difference between the two groups is statistically significant (*P* < 0.05).

**FIGURE 5:**
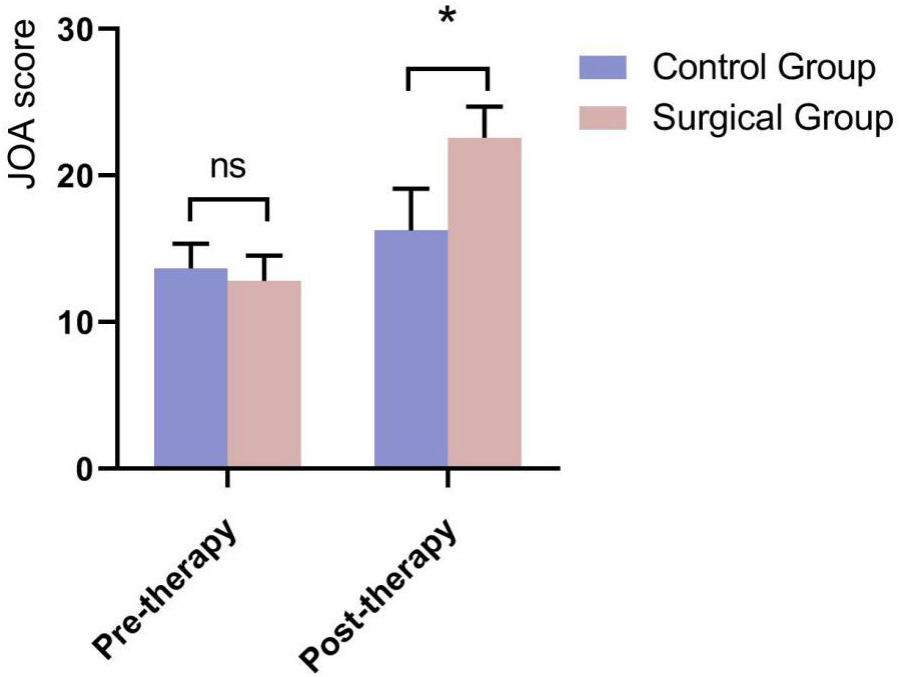
Comparison of JOA scores 1 week postoperatively between the two groups. *Indicates that the difference between the two groups is statistically significant (*P* < 0.05).

A comparison of Visual Analog Scale (VAS) scores at different time points, as well as pre- and post-treatment ODI (Oswestry Disability Index) and JOA (Japanese Orthopedic Association) scores between the two groups, showed no significant differences in pre-treatment VAS, ODI, and JOA scores between the groups (*P* > 0.05). However, for the Intervention group, the VAS scores at 1 week, 1 month, 6 months, and 12 months postoperatively were significantly lower than those in the Control group, with statistically significant differences (*P* < 0.05) (Figure 3 and Table 2). The between-group mean difference in VAS score at 12 months was –1.98 points (95% CI: –2.42 to –1.53), favoring the Intervention group. Postoperatively, the ODI scores in the Intervention group were significantly lower than those in the Control group. The between-group mean difference in ODI score at 1 week post-treatment was –7.88 points (95% CI: –8.80 to –6.96), favoring the Intervention group. While the JOA scores were significantly higher, with statistically significant differences (*P* < 0.05) (Figures 4, 5 and Table 3). The between-group mean difference in JOA score at 1 week post-treatment was +6.33 points (95% CI: 5.39–7.27), favoring the Intervention group.

**TABLE 2:** Comparison of VAS scores at different time points postoperatively between the two groups of patients ($\bar{x}$ ± s).

Group	VAS score				
	Pre-therapy	Post 1 week	Post 1 month	Post 6 month	Post 12 month
Control group (*n* = 342)	5.57 ± 1.307	3.07 ± 1.326	3.67 ± 0.933	4.18 ± 1.444	4.23 ± 1.407
Intervention group (*n* = 215)	5.89 ± 1.329	2.15 ± 0.951	1.80 ± 0.730	2.07 ± 0.979	2.25 ± 0.947
*t*-value	1.319	–4.247	–11.874	–9.093	–8.768
*P*-value	0.758	0.023	0.028	0.005	0.003

**TABLE 3:** Comparison of ODI scores and JOA scores 1 week postoperatively between two groups of patients ($\bar{x}$ ± s).

Group	ODI score		JOA score	
	Pre-therapy	Post 1 week	Pre-therapy	Post 1 week
Control group (*n* = 342)	35.72 ± 4.618	22.12 ± 2.871	13.67 ± 1.684	16.25 ± 2.856
Intervention group (*n* = 215)	36.78 ± 4.135	14.24 ± 1.990	12.80 ± 1.736	22.58 ± 2.132
*t*-value	1.299	–16.959	–2.716	13.376
*P*-value	0.633	0.012	0.865	0.034

### Clinical outcomes

The excellent and good rate of the Control group patients based on the modified MacNab Score 1 year postoperatively was 35.09%, while the Intervention group was 77.67%. The difference between the two groups of patients was statistically significant (*P* < 0.05) (Table 4).

**TABLE 4:** Comparison of clinical efficacy 1 year postoperatively between two groups of patients ($\bar{x}$ ± s).

Group	*N*	Modified MacNab score/n				Excellent and good rate/%
		Excellent	Good	Fair	Poor	
Control group	342	51	69	184	38	35.09
Intervention group	215	94	73	32	16	77.67
*χ^2^*-value						105.308
*P*-value						0.000

### Complications and drug side effects

Adverse events were actively monitored at each follow-up visit and recorded prospectively in the institutional electronic medical record system. In the Control group, 26 patients experienced symptoms such as stomach discomfort, acid reflux, mild stomach pain, and poor appetite. After receiving symptomatic treatment such as acid suppression and stomach protection, their symptoms were alleviated, and the symptoms disappeared after discontinuation of the medication. In the Intervention group, 3 patient experienced postoperative chest tightness, which improved spontaneously after oxygen therapy and electrocardiographic monitoring. Additionally, 4 patients developed numbness in the nerve-innervated area. No complications such as infection, bleeding, or hypotension were observed.

## Discussion

The mechanism underlying lower limb neuropathic pain caused by lumbar disc herniation (LDH) has not been fully elucidated. Due to the degeneration of the lumbar intervertebral disc, rupture of the annulus fibrosus, and protrusion of the nucleus pulposus, the protruded disc tissue mechanically compresses the nerve root, leading to obstruction of the blood circulation around the nerve root, increased capillary permeability, and leakage of inflammatory pain-inducing substances (4, 6). This irritation provokes the nerve root and its surrounding tissues. This stimulation initiates sterile inflammation in the nerve root and its surrounding tissues, further causing a series of symptoms such as low back and leg pain, and radiating sciatic nerve pain in the lower limbs. Therefore, addressing the inflammatory response of the nerve root is one of the important directions in the treatment of LDH in pain management (33, 34).

Pulsed radiofrequency (PRF) treatment utilizes high-frequency pulsed currents emitted by a radiofrequency generator to generate heat through the friction of ion movement within the target tissue. This selectively damages the conductive branches of nociceptive nerve fibers, blocking the transmission of pain signals to the superior nerves and disrupting the pain conduction pathway, thereby achieving the purpose of pain control (35, 36). In contrast, selective nerve root block involves injecting local anesthetics and steroidal drugs around the nerve root (27). Local anesthetics can reduce the afferent pain sensation of inflamed tissues, while steroidal drugs inhibit prostaglandin synthesis to achieve anti-inflammatory and immunosuppressive effects. Among them, dexamethasone palmitate can be quickly absorbed through capillaries, phagocytosed by macrophages, and slowly releases dexamethasone active substances after lipase hydrolysis (31). Compared to traditional water-soluble dexamethasone, dexamethasone palmitate offers higher safety and more prolonged anti-inflammatory effects. In comparison, orally administered drugs primarily act on the entire body after oral absorption, and their effectiveness is influenced by various factors such as drug absorption, distribution, metabolism, and excretion. Additionally, oral medications often have associated side effects.

In our hospital’s clinical practice, the combination of pulsed radiofrequency (PRF) and dexamethasone palmitate selective nerve root block for the treatment of LDH has achieved favorable clinical outcomes. Through rigorous clinical comparative trials, we have found that PRF combined with dexamethasone palmitate selective nerve root block is superior to oral medications in alleviating pain, improving quality of life, and promoting lumbar function recovery. Importantly, the observed improvements in VAS and ODI scores exceeded commonly reported minimal clinically important difference (MCID) thresholds (approximately 1.5–2 points for VAS and 8–12 points for ODI), suggesting that the differences between groups were not only statistically significant but also clinically meaningful. These findings are consistent with multiple research results both domestically and internationally. Wu et al. (37) suggested that PRF can significantly relieve inflammatory pain by inhibiting neuroinflammation through reducing NF-κB phosphorylation levels, and is an economical, safe, and effective minimally invasive interventional technique. Gazioğlu et al. (38) believed that PRF exhibits significant analgesic effects, and for patients with short-duration lumbar pain, the combined application of continuous medial branch radiofrequency ablation of the facet joint can enhance therapeutic efficacy. Han et al. (39) conducted epidural injections with different doses of dexamethasone palmitate, confirming its reliable safety and effectiveness in improving pain symptoms and functional impairments in patients with LDH. However, some studies have also suggested that oral medications may still have certain advantages (40) in certain situations, which may be related to individual patient differences, severity of the disease, and medication choices.

### Limitations

Certainly, this study has certain limitations. First, as a retrospective analysis, it is inherently subject to selection bias and information bias, which may affect the reliability of the findings. Second, despite the lack of statistically significant differences in baseline characteristics between groups, the non-randomized design may still introduce treatment-selection bias, as the choice of therapy could have been influenced by the physician’s judgment or the patient’s condition. Third, the study is a single-center investigation, which may limit the generalizability of our conclusions to broader populations with different demographic or clinical characteristics. Fourth, the sample size is relatively modest, which may not fully capture the heterogeneity of all patients with lumbar disc herniation. Fifth, the Control group received oral diclofenac sodium rather than an interventional placebo or a standardized alternative interventional therapy, making it difficult to definitively isolate the specific therapeutic effect of the pulsed radiofrequency and selective nerve root block. Finally, the study duration is relatively short, and the long-term efficacy of pulsed radiofrequency combined with dexamethasone palmitate selective nerve root block has not been observed. In the future, well-designed, multicenter, randomized controlled trials with larger sample sizes and extended observation periods are necessary to validate and build upon the findings of this study.

At the same times, we acknowledge that the treatment intensity and invasiveness differed substantially between the two groups, with the Intervention group receiving a combined interventional procedure and the Control group receiving only oral medication. This difference reflects the real-world clinical scenario in our institution: patients with mild to moderate symptoms typically receive conservative pharmacological management, while those with more pronounced radicular pain or insufficient response to medication are more likely to undergo interventional procedures after shared decision-making. Although statistical analysis showed no significant differences in baseline characteristics (including pain scores), we recognize that unmeasured confounders such as patient preference, symptom duration acuity, or psychological factors may have influenced treatment selection. Therefore, while the comparison offers valuable insights into the relative effectiveness of two distinct treatment strategies, the findings should be interpreted with caution due to the inherent treatment-intensity bias in this non-randomized comparison. Because treatment allocation was not randomized and was based on real-world clinical decision-making, residual confounding cannot be excluded despite comparable baseline characteristics.

## Conclusion

In summary, pulsed radiofrequency combined with dexamethasone palmitate selective nerve root block therapy was associated with improved pain relief and functional recovery compared to oral medication in this retrospective cohort. Its characteristics of direct targeting, strong pertinence, and minimal side effects suggest potential clinical utility in appropriately selected patients. However, given the significant differences in treatment intensity between the groups and the non-randomized study design, these findings should be considered preliminary. Well-designed randomized controlled trials with comparable control interventions (e.g., sham procedures or standardized conservative therapy) are needed to confirm the superior efficacy of this combined interventional approach.

## Data Availability

The original contributions presented in this study are included in this article/supplementary material, further inquiries can be directed to the corresponding author.
